# Modulation of Notch Signaling Pathway by Bioactive Dietary Agents

**DOI:** 10.3390/ijms23073532

**Published:** 2022-03-24

**Authors:** Violet A. Kiesel, Silvia D. Stan

**Affiliations:** 1 Department of Nutrition Science, Purdue University, West Lafayette, IN 47907, USA; vkiesel@purdue.edu; 2 Department of Nutrition, College of Agriculture, Biotechnology and Natural Resources, University of Nevada, Reno, NV 89557, USA

**Keywords:** Notch signaling, dietary agents, chemoprevention, cancer stem cells

## Abstract

Notch signaling is often aberrantly activated in solid and hematological cancers and regulates cell fate decisions and the maintenance of cancer stem cells. In addition, increased expression of Notch pathway components is clinically associated with poorer prognosis in several types of cancer. Targeting Notch may have chemopreventive and anti-cancer effects, leading to reduced disease incidence and improved survival. While therapeutic agents are currently in development to achieve this goal, several researchers have turned their attention to dietary and natural agents for targeting Notch signaling. Given their natural abundance from food sources, the use of diet-derived agents to target Notch signaling offers the potential advantage of low toxicity to normal tissue. In this review, we discuss several dietary agents including curcumin, EGCG, resveratrol, and isothiocyanates, which modulate Notch pathway components in a context-dependent manner. Dietary agents modulate Notch signaling in several types of cancer and concurrently decrease in vitro cell viability and in vivo tumor growth, suggesting a potential role for their clinical use to target Notch pathway components, either alone or in combination with current therapeutic agents.

## 1. Introduction

The Notch signaling pathway is a key developmental pathway that regulates many cellular processes, including cell proliferation, differentiation, and maintenance of cancer stem cells (CSC) [1,2]. The Notch signaling pathway is active in embryonic development, and aberrant Notch signaling has been identified in several types of cancer, including T-cell acute lymphoblastic leukemia (T-ALL), breast, lung, and glioma, among others [3,4]. In adults, Notch maintains tissue-specific stem cell populations, such as those found in the intestinal crypts, through the process of self-renewal [5]. By extension, aberrantly activated Notch maintains the CSC population in transformed cells [6]. Clinical data show inverse associations between the expression of Notch pathway components and patient survival in several types of cancer, correlating Notch activation with aggressiveness of cancer [3,7,8,9]. Several therapeutic agents have been designed to target the Notch pathway, although the safety and efficacy of these agents are still being evaluated [4,6,10]. Several researchers have focused their attention on the identification of dietary or natural agents which modulate Notch signaling. This review discusses the efficacy of several dietary and natural agents in the regulation of the Notch signaling pathway in the context of cancer.

## 2. Notch Signaling

Notch signaling is activated by direct contact between Notch ligands and receptors expressed on opposing cells. Five single-pass transmembrane Notch ligands (Jagged-1, Jagged-2, and Delta-like (DLL)-1, -3, and -4) have been identified in mammals. Notch ligands activate one of four membrane-bound Notch receptors (Notch-1, -2, -3, -4). Contact between a ligand and a receptor induces a S2 cleavage of the Notch receptor by a disintegrin and metalloprotease (ADAM)-10, liberating the Notch extracellular domain (NECD), which is endocytosed into the ligand-expressing cell [1,5,11]. Notch can also be activated by ligand-independent mechanisms [12]. ADAM-17 preferentially performs S2 cleavage under ligand-independent conditions [13].

The cleavage of Notch by ADAM-10 or ADAM-17 is followed by an S3/S4 cleavage of Notch by the gamma secretase complex [14]. Gamma secretase cleavage liberates the Notch intracellular domain (NICD). NICD translocates to the nucleus, where it binds the transcription factor CSL that represents CBF-1/RBPJ-κ (recombinant signal binding protein for immunoglobulin kappa J region) in mammals [1]. The binding of NICD to RBP-Jκ induces the recruitment of mastermind-like protein (MAML) and the transcriptional activation complex, thereby activating transcription [11,15]. Classic Notch downstream targets include Hes and Hey family members, which are frequently used as markers of pathway activation.

Of particular interest in cancer biology is the role of Notch in regulation of the CSC population. CSCs express detoxifying and drug efflux proteins such as ATP-binding cassette subfamily G2 (ABCG2) and aldehyde dehydrogenase (ALDH), which confer treatment resistance [16]. The survival of CSCs following treatment permits tumor regrowth and subsequent patient relapse [17]. The strength of evidence that Notch contributes to CSC maintenance is high. Breast cancer cells with high Notch pathway activity demonstrate enhanced sphere-formation capacity in vitro and tumor-formation capacity in vivo compared to cells with low Notch activity [18]. In accordance with this, knock-down or overexpression of Notch inhibits or enhances sphere formation, respectively, in cancer cells [19,20].

In addition to its direct role in regulating CSCs and epithelial to mesenchymal transition (EMT), the Notch pathway cross-talks with several pathways including Ras, Wnt, nuclear factor kappa B (NF-κB), Janus Kinase/Signal Transducer and Activator of Transcription (JAK/STAT) signaling and others, which contribute to cancer cell proliferation [21]. The simultaneous overexpression of Ras and Notch-1 induced the malignant transformation of HMLE human mammary epithelial cells, while the overexpression of either of these two genes alone did not result in transformation, suggesting a cooperative relationship between the pathways [22]. The Notch ligand Jagged-1 is a transcriptional target of Wnt signaling pathway [23]. In addition, β-catenin, a component of Wnt signaling pathway, can directly bind NICD in HEK293 human embryonic kidney cells, and the overexpression of β-catenin increases Hes-1 reporter activity in mouse embryonic fibroblasts [24]. These data suggest that Wnt activation can augment Notch pathway activity. Both NF-κB and JAK/STAT signaling cross-talk with Notch in a bi-directional manner [25]. NF-κB is a Notch target gene [3], suggesting that Notch inhibition may also result in NF-κB pathway deactivation. In addition, Notch is a regulator of angiogenesis in cancer cells [26]. Collectively, these data indicate that the modulation of Notch may alter the activity of several other pathways relevant to tumor progression.

Notch signaling regulates gene expression in a cell-type- and context-dependent manner [2,3,11]. Notch has been shown to have both an oncogenic role and a tumor-suppressive role in different cancer cells, depending on the cellular context [2,10]. Therefore, Notch mutations are either activating or inactivating, in function of the overall role that Notch plays in that specific cellular context as either an oncogene or a tumor suppressor [10].

Several approaches to modulating Notch signaling in cancer are actively being researched, including the use of monoclonal antibodies and gamma secretase inhibitors (GSIs) [4,10]. GSI use in cancer, while sometimes efficacious in tumor remission, is associated with undesirable side effects, including diarrhea, nausea, and vomiting [4,6]. Given the crucial role of Notch signaling in normal tissue, an ideal therapeutic agent would specifically target Notch signaling in cancer cells, while leaving Notch in the normal tissue unaffected. The use of diet-derived agents, either alone or in combination with current chemotherapeutic agents, may be a possible route for targeting Notch in cancer cells with minimal toxicity to the surrounding healthy tissue.

## 3. Modulation of Notch Pathway by Dietary Agents

The appeal of diet-derived solutions for the prevention of cancer originated from epidemiological data suggesting that certain populations may have a lower cancer risk based on their dietary patterns. Correlations have been identified, for example, between the Mediterranean diet and a reduced cancer risk [27]. Reductionist examinations of diet patterns indicate that specific foods and food bioactive compounds underlie the reduced risk of disease development, suggesting that these bioactive compounds could be used in chemopreventive or as adjuvant treatment-focused models.

### 3.1. Curcumin

**Figure 1:**
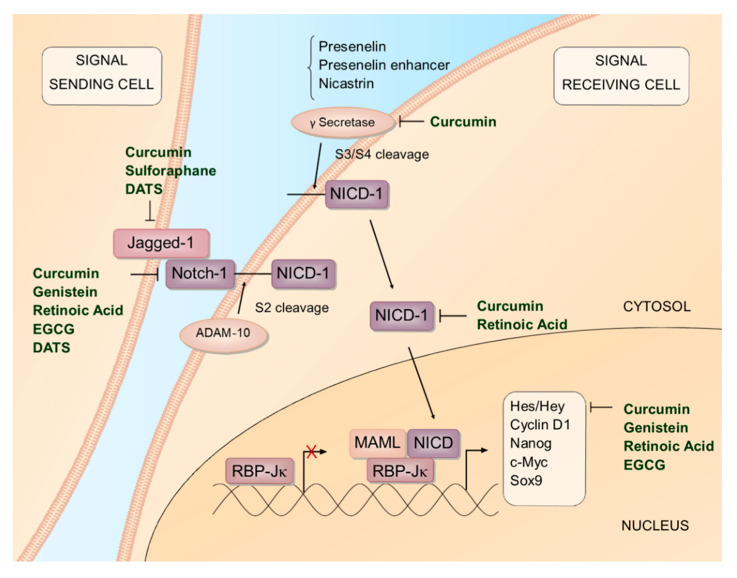
Effect of dietary agents on Notch signaling pathway in cancer cells. Notch ligands, including Jagged-1, bind to, and permit cleavage of, the Notch receptor. The Notch extracellular domain is first cleaved by ADAM-10; Notch intracellular domain (NICD) is then cleaved by the gamma secretase complex. NICD translocates and activates transcription in the nucleus. Dietary agents that modulate Notch in cancer cells in vitro are listed. EGCG, epigallocatechin-3-gallate; DATS, diallyl trisulfide; RBP-Jκ, recombinant signal binding protein for immunoglobulin kappa J region; MAML, mastermind-like protein.

Curcumin, the bright yellow bioactive compound derived from *Curcuma longa* species plants, and its analogs, reduced the expression of Notch pathway components including Notch-1, Jagged-1, components of the gamma secretase complex, and Notch downstream targets in several cancer models (Figure 1, Table 1) [28,29,30,31,32,33,34,35]. Curcumin analogs or enhanced methods of delivery are preferred given the low bioavailability of curcumin from its dietary source, turmeric [36]. In a xenograft mouse model with HCT116 human colon cancer cells, oral administration of the turmeric/phospholipid formulation Meriva^®^, in combination with oxaliplatin treatment, inhibited NICD-1 in tumor tissue [29], suggesting that curcumin may improve the efficacy of anticancer drugs (Table 2). Curcumin modulates the expression of pathways and processes downstream of Notch. In U2OS osteosarcoma cells, curcumin inhibits the expression of Notch-1 and matrix metalloproteases (MMP)-2, and -9 [31]. Importantly, curcumin treatment also inhibits invasion in U2OS cells, and invasion is rescued by the overexpression of Notch-1 [31].

### 3.2. Genistein

Genistein is a polyphenolic, soy-derived isoflavone. Mammary epithelial cells from rats fed lifelong diets including soy protein isolate or genistein supplementation had reduced Notch-2 mRNA compared to casein-fed rats, although whether this inhibitory effect translates to a chemopreventive effect cannot be determined [65]. In breast, colon, and neuroblastoma cancer cell lines, genistein suppressed Notch-1 protein expression (Table 2) [44,45,46]. In breast cancer cells, the suppression of Notch-1 by genistein coincides with the downregulation of cyclin B1 and Bcl-2, and this effect is mimicked by Notch-1 siRNA treatment [44]. Further research is required to determine if cell cycle inhibition and apoptosis induction by genistein are a direct downstream consequence of Notch inhibition.

### 3.3. EGCG and Tea Polyphenols

The green tea polyphenol epigallocatechin-3-gallate (EGCG) modulates the expression of Notch pathway components in vitro (Table 1). EGCG inhibited Notch-1 in neuroblastoma, cholangiocarcinoma, and colon cancer cell lines, and in head and neck squamous cell carcinoma CSCs [41,42,66,67]. EGCG inhibited Notch-2 in colon cancer cell line [40], and nearly ablated Hes-1 gene expression in colon cancer cells, suggesting pathway deactivation. The EGCG analog theaflavin-3,3’-digallate (TF3) inhibited NICD-1 expression in ovarian cancer OVCAR-3 cells [68]. In squamous cell carcinoma of the tongue cell lines, treatment with EGCG modulated Notch-4 expression, although the direction of this effect (up or down) varied by EGCG incubation time and cell line [69].

EGCG and its related compounds modulate Notch-related pathways and processes, including angiogenesis, EMT, and maintenance of a CSC phenotype. TF3 inhibited c-Myc, hypoxia-inducible factor 1α (HIF-1α), and VEGF in ovarian cancer cells [68]. The inhibition of c-Myc, HIF-1α, and VEGF by TF3 was reversed by ectopic expression of NICD-1 [68]. EGCG induced E-cadherin and suppressed MMP-2 and -9 in neuroblastoma cells [42]. EGCG-mediated Notch inhibition reduced sphere formation in head and neck carcinoma cells, and this effect was augmented by the addition of cisplatin [41]. Furthermore, pre-treatment of head and neck CSCs with cisplatin, either alone or in combination with EGCG, prior to grafting into BALB/c nude mice, inhibited tumor growth in both models, and the effect was strongest with co-treatment [41]. In a K-Ras transgenic mouse model, EGCG was shown to inhibit tumoral lesions on lip and tongue by down-regulation of the Notch pathway [43]. Taken together, these data support the modulation of Notch receptors and activity by EGCG, as well as a role for EGCG in reducing the markers of an aggressive cancer phenotype.

### 3.4. Resveratrol

Studies of resveratrol, the stilbene found in the skins of grapes, peanuts, and blueberries, underscore the context-dependent nature of Notch signaling as both oncogenic- and tumor-suppressive in cancer cells (Table 1). Accumulating evidence suggests that certain types of cancer, including neuroendocrine tumors and brain cancers, rely on Notch activation for tumor suppression [70,71].

Carcinoid tumors are slowly growing neuroendocrine tumors that usually grow in the gastrointestinal tract or lungs. In cultured neuroendocrine cancer cells from gastrointestinal and pulmonary carcinoids, resveratrol treatment induced activation of Notch signaling, as indicated by Notch-2 induction and the suppression of achaete-scute complex-like 1 (ASCL-1), a downstream target of Notch. In addition, resveratrol suppressed expression of neuroendocrine hormones and reduced carcinoid proliferation in vitro and in vivo [48].

Resveratrol increased Notch-2 mRNA, induced apoptosis and suppressed neuroendocrine marker ASCL-1 in medullary thyroid cancer (MTC) [52]. In anaplastic thyroid carcinoma (ATC), resveratrol activated Notch-1 signaling and suppressed growth of ATC cells in vitro and in vivo [72]. Taken together, these data support a tumor-suppressive role of Notch in MTC and ATC [52,72].

In addition, resveratrol induced Notch-1 in glioblastoma cells while the Notch-1 inhibitor MRK-003 partially reversed the resveratrol-mediated inhibition of proliferation, supporting a tumor-suppressive role of Notch in glioblastoma [49]. In medulloblastoma cells, resveratrol induction of Notch-1 and Notch-2 had a minimal effect on meduloblastoma cell growth [73].

Resveratrol suppressed Notch in cell models of T-ALL, cervical, ovarian, and breast cancer [47,50,51,74]. Culturing ovarian and cervical cancer cells with a GSI caused a decrease in Hes-1 protein expression without altering viability, suggesting that Notch inhibition is dispensable to cancer cell death [47,50]. These data collectively underscore the context-dependent nature of Notch signaling.

### 3.5. Retinoic Acid

Retinoic acid and related retinoids inhibit Notch expression and signaling in cultured cancer cells (Figure 1, Table 1). All-trans retinoic acid (ATRA) inhibited Notch-1 expression in glioblastoma, breast and ovarian cancer cells, and NICD-1 in glioblastoma cells [54,56,75]. ATRA suppressed Notch-3 protein in MDA-MB-231 breast cancer cells [53]. The retinoids 4-HPR, Cl-AHPC, and AHP3 inhibited Notch-1 in neuroblastoma [55] and pancreatic cancer cell lines [57].

The retinoid 4-HPR induced E-cadherin expression in neuroblastoma cells [55]. In contrast, neuroblastoma cells treated with 13-cis retinoic acid displayed increased migration compared to untreated cells in a Notch-independent manner. However, treatment of 13-cis retinoic acid induced cell-cycle arrest and increased the fraction of Annexin-V-positive cells [76]. Historically, ATRA has been used as a differentiation-inducing agent [77]. Treatment with ATRA reduced sphere size and formation in glioblastoma and ovarian cancer cells, and inhibited ALDH-1 in ovarian cancer cells [54,56]. Pre-treating glioblastoma and ovarian cancer cells with ATRA prior to grafting into SCID mice impaired tumor formation, suggesting that ATRA reduces the tumorigenic capacity of cancer cells [54,56]. Furthermore, in a glioblastoma xenograft model, dissociated ATRA-treated tumors displayed reduced sphere formation when cultured ex vivo compared to untreated dissociated tumor cells [54]. The retinoids Cl-AHPC and AHP3 also blocked sphere formation in pancreatic cancer cells [57]. These data support an anti-CSC role for ATRA in vitro and in vivo.

### 3.6. Sulforaphane

Sulforaphane, an isothiocyanate derived from cruciferous vegetables, inhibited full-length Notch-1, -2, and -4 protein expression in prostate cancer cells, while upregulating the expression of NICD-1, -2, and -4 [63]. Hes-1 reporter activity was induced by sulforaphane in LNCaP and PC-3 prostate cancer cells [63]. Tumor tissue from transgenic adenocarcinoma mouse prostate mice treated with sulforaphane, in contrast, had reduced NICD-2 expression compared to control-treated animals [63] (Table 2). Sulforaphane treatment inhibited prostate cancer cell migration and the expression of EMT-related proteins, although this effect was Notch-independent [61,63]. Sulforaphane inhibited Notch-1, and negated a gemcitabine-induced rise in Notch-1 expression in pancreatic cancer cells cultured in vitro [60]. Sulforaphane inhibited tumor growth in xenograft nude mice when MiaPaCa2 pancreatic cancer cells were pre-treated with the agent before grafting, and when sulforaphane was administered after grafting [60], suggesting chemopreventive and anticancer roles of sulforaphane in vivo. Further studies are required to determine the effect of sulforaphane on Notch in preclinical in vivo models of pancreatic cancer.

### 3.7. Vitamin D

Results regarding the efficacy of vitamin D as a Notch modulator are mixed. Treatment with 1,25-dihydroxyvitamin D3 (1,25(OH)2D3) had no effect on Notch-2, Notch-4, or Jagged-1 protein expression in a cell model of glioblastoma, or on Notch-1 or Jagged-1 in keratinocytes [78,79]. In the MCF10DCIS.com breast cancer cells, the Gemini vitamin D analog BXL0124 blocked Notch-1 activation and the expression of Jagged-1, -2, and DLL-1 (Table 1) [39]. Conversely, BXL0124 rapidly induced the message and protein expression of Hes-1 in MCF10DCIS.com cells [39]. Knock-down of Hes-1 with siRNA partially reversed the BXL0124-mediated suppression of NICD-1, Jagged-2, and c-Myc protein. Hes-1 overexpression in the absence of BXL0124 suppressed NICD-1, Jagged-2, and c-Myc, suggesting a negative feedback loop in which Hes-1 inhibits Notch activation. In the SUM159 triple-negative breast cancer cells, the vitamin D compounds down-regulated Notch-1, Notch-2, Notch-3, Jagged-1, Jagged-2, and Hes-1 [80]. Tumor tissue from SKOV-3 xenograft nude mice i.p. treated with the vitamin D analog MT19c had decreased expression of Notch pathway components, and increased DNA fragmentation relative to control (Table 2) [64]. Further studies on the role of vitamin D and its analogs on Notch modulation across several types of cancer are warranted.

### 3.8. Other Agents

Honokiol, a traditional Chinese and Japanese herbal therapeutic, inhibited Notch pathway components, including Notch-1 and -2, Jagged-1, Hes-1, and subunits of the gamma secretase complex in melanoma, hepatocellular carcinoma, and colon cancer cells [20,62,81,82]. In addition, sphere formation and cell survival were inhibited by honokiol treatment, alone or in combination with a single dose of ionizing radiation at 5 Gy [20,81]. Honokiol in combination with ionizing radiation inhibited NICD-1, Jagged-1, Hes-1, members of γ secretase complex, and tumor growth in vivo (Table 2) [62].

Withaferin A, a lactone found in the leaves of *Withania somnifera* (Indian Winter cherry) suppressed NICD-1 and induced NICD-2 and NICD-4 expression and RBP-Jκ reporter activity in breast cancer cells [83]. Withaferin A suppressed ALDH1 activity, although in a Notch-independent manner [84]. Withaferin A inhibited NICD-1 in SKOV3 ovarian cancer cells and colon cancer cell lines [85,86]. Importantly, the viability of the normal colon cell line FHC was not affected by withaferin A treatment [86], suggesting low toxicity towards normal tissue. In A2780 ovarian cancer cells, withaferin A inhibited Notch-1 and enhanced the therapeutic effect of Doxil, a liposomal preparation of doxorubicin [87].

Hesperetin, a flavonoid found in citrus fruits, activates Notch-1 signaling, and induced apoptosis and expression of differentiation markers in ATC cells [88].

Phenethyl isothiocyanate (PEITC), a cruciferous-vegetables-derived isothiocyanate, suppressed Notch-1 and Notch-2 levels, reduced cell proliferation, and induced apoptosis in pancreatic cancer cells [59]. In HER2-positive breast and ovarian carcinoma cells, PEITC decreased the expression of NICD-1 and targeted both differentiated cells and CSCs [58].

Diallyl trisulfide (DATS), a dietary bioactive compound derived from *Allium* vegetables, inhibited the expression of Notch-1, and Hes-1 in osteosarcoma cells [37]. DATS suppressed Notch ligands Jagged-1 and Jagged-2 in MDA-MB-231 and MCF-7 breast cancer cells, and in Harvey-ras (H-ras) transformed MCF10A-H-Ras breast epithelial cells [38]. In addition, DATS inhibited alpha-secretases ADAM-10 and ADAM-17 in breast cancer cells [38], supporting a role for DATS in the inhibition of Notch pathway components often overexpressed in breast tumors.

## 4. Conclusions

In summary, the Notch signaling pathway plays a key role in the development and progression of cancer. Targeting Notch is also important due to the pathway’s extensive degree of cross-communication with other signaling pathways, including Wnt, NF-κB, and JAK/STAT, which confer survival advantages to cancer cells. In addition, Notch activation is critically involved in the maintenance of highly tumorigenic and treatment-resistant CSCs, which play a role in tumor survival and recurrence. Several dietary agents including curcumin, EGCG, resveratrol, and isothiocyanates have been shown to modulate Notch pathway components in a context-dependent manner. Further studies are needed to identify causal relationships between the modulation of Notch components by dietary agents and tumor cell growth and metastasis. In addition, future studies characterizing the role of dietary agents, alone or in combination with chemotherapeutic agents, on the modulation of Notch signaling will further our understanding of Notch biology and improve cancer prevention and treatment.

## Figures and Tables

**Table 1:** Effect of dietary agents on Notch pathway components in vitro by cancer type.

		↑	Induced	↓	Suppressed				
Dietary Agent	Cancer Type		Notch-1	Notch-2	Notch-3	Jagged-1	Hes-1	Hey-1	References
*Curcumin*	Cholangiocarcinoma		↓				↓		[30]
	Colorectal *^, #^		↓		↓				[29,32]
	Esophageal		↓			↓	↓		[33]
	Lymphoma		↓						[28]
	Osteosarcoma		↓				↓	↓	[31]
	Prostate		↓			↓			[34]
*DATS*	Osteosarcoma		↓				↓		[37]
	Breast					↓			[38]
*Vitamin D*	Breast *		↓		↓	↓	↑		[39]
*EGCG*	Colorectal		↑	↓			↓		[40]
	Head and Neck		↓						[41]
	Neuroblastoma		↓						[42]
	Tongue		↓	↓			↓		[43]
*Genistein*	Breast		↓						[44]
	Colon		↓						[45]
	Neuroblastoma		↓				↓		[46]
*Resveratrol*	Cervical		↓	↓			↓		[47]
	Carcinoids		↑						[48]
	Glioblastoma		↑						[49]
	Ovarian			↓			↓		[50]
	T-ALL		↓				↓		[51]
	Thyroid		↑	↑					[52]
*Retinoic Acid*	Breast				↓				[53]
	Glioblastoma		↓					↓	[54]
	Neuroblastoma *		↓						[55]
	Ovarian		↓						[56]
	Pancreatic *		↓						[57]
*PEITC*	Breast		↓				↓		[58]
	Pancreatic		↓	↓					[59]
*Sulforaphane*	Pancreatic		↓						[60]
	Prostate		↓			↓			[61]

* Indicates use of dietary analog; ^#^ indicates alternate delivery method e.g., nanoparticle; DATS, diallyl trisulfide; EGCG, epigallocatechin-3-gallate; PEITC, phenethyl isothiocyanate.

**Table 2:** Effect of dietary agents on Notch pathway components in vivo by cancer type.

				↑	Induced	↓	Suppressed	
Dietary Agent	Concentration	Cancer Type	Effect					References
*Curcumin*	0.2% equivalent curcuminoids as Meriva^®, #^	Colorectal	↓	NICD-1, Ki67				[29]
			↑	cleaved Caspase 3				
			↓	tumor volume; additive effect on tumor volume with oxaliplatin				
*Honokiol*	200 µg/kg body weight	Colorectal	↓	NICD-1, Jagged-1, Hes-1, Presenilin-1, Nicastrin				[62]
			↓	tumor volume				
*Sulforaphane*	6 µmol SFN in 0.1 mL PBS	Prostate	↓	NICD-2				[63]
*EGCG*	25 mg/kg body weight	Lip, Tongue	↓	Notch-1, Notch-2, Hes1				[43]
*Vitamin D*	10 mg/kg body weight of MT19c *	Ovarian	↓	Notch signaling pathway				[64]
			↑	DNA fragmentation				
			↓	tumor volume				

^#^ Indicates alternate delivery method e.g., nanoparticle; Meriva^®^, turmeric/phospholipid formulation; * Indicates use of dietary analog; MT19c, vitamin D analog; SFN, sulforaphane; PBS, phosphate buffer saline; NICD, Notch intracellular domain.
